# Visualization of medical concepts represented using word embeddings: a scoping review

**DOI:** 10.1186/s12911-022-01822-9

**Published:** 2022-03-29

**Authors:** Naima Oubenali, Sabrina Messaoud, Alexandre Filiot, Antoine Lamer, Paul Andrey

**Affiliations:** 1 Faculté Ingénierie et Management de la Santé, Univ. Lille, 59000 Lille, France; 2 INCLUDE: Integration Center of the Lille University Hospital for Data Exploration, CHU Lille, 59000 Lille, France; 3 ULR 2694 - METRICS: Évaluation des Technologies de Santé et des Pratiques Médicales, CHU Lille, Univ. Lille, 59000 Lille, France

**Keywords:** Word embeddings, Visualization, Data mining, Medical, Natural language processing, Deep learning

## Abstract

**Background:**

Analyzing the unstructured textual data contained in electronic health records (EHRs) has always been a challenging task. Word embedding methods have become an essential foundation for neural network-based approaches in natural language processing (NLP), to learn dense and low-dimensional word representations from large unlabeled corpora that capture the implicit semantics of words. Models like Word2Vec, GloVe or FastText have been broadly applied and reviewed in the bioinformatics and healthcare fields, most often to embed clinical notes or activity and diagnostic codes. Visualization of the learned embeddings has been used in a subset of these works, whether for exploratory or evaluation purposes. However, visualization practices tend to be heterogeneous, and lack overall guidelines.

**Objective:**

This scoping review aims to describe the methods and strategies used to visualize medical concepts represented using word embedding methods. We aim to understand the objectives of the visualizations and their limits.

**Methods:**

This scoping review summarizes different methods used to visualize word embeddings in healthcare. We followed the methodology proposed by Arksey and O’Malley (Int J Soc Res Methodol 8:19–32, 2005) and by Levac et al. (Implement Sci 5:69, 2010) to better analyze the data and provide a synthesis of the literature on the matter.

**Results:**

We first obtained 471 unique articles from a search conducted in PubMed, MedRxiv and arXiv databases. 30 of these were effectively reviewed, based on our inclusion and exclusion criteria. 23 articles were excluded in the full review stage, resulting in the analysis of 7 papers that fully correspond to our inclusion criteria. Included papers pursued a variety of objectives and used distinct methods to evaluate their embeddings and to visualize them. Visualization also served heterogeneous purposes, being alternatively used as a way to explore the embeddings, to evaluate them or to merely illustrate properties otherwise formally assessed.

**Conclusions:**

Visualization helps to explore embedding results (further dimensionality reduction, synthetic representation). However, it does not exhaust the information conveyed by the embeddings nor constitute a self-sustaining evaluation method of their pertinence.

## Background

The broad adoption of electronic health records (EHRs) has generated high volumes of digital clinical data, comprising both structured information, such as biology results or diagnostic codes, and unstructured information, most notably in the form of textual data [1]. The latter can be explored, analyzed and modeled using Natural Language Processing (NLP) and Machine Learning techniques [2, 3]. A key challenge that has gathered substantial efforts from the medical informatics community is to create structured representations of the unstructured data that can make it more understandable, and potentially ease their use in downstream modeling tasks. Among other approaches, word embedding methods have become a major reference to tackle this issue [4].

Over the last decade, word embeddings have become an essential foundation for neural network-based approaches in NLP to effectively learn dense and low-dimensional word embeddings from large unlabeled corpora that capture the implicit semantics of words [5–7]. They consist of learning a model (often a single matrix) that replaces individual words with numerical vectors of fixed dimensionality whose relative geometrical positions reflect and highlight similarity properties of the embedded words. Those embeddings are most often learned in an unsupervised or self-supervised way, *i.e.* without using any prior or expert labeling of the data. Once learned, embeddings are often transferred to a supervised learning problem, *i.e.* they are re-used to generate structured input representations for classification or regression models. Whether in a medical context or not, this transfer learning approach has been demonstrated to yield significant performance improvements for a multitude of downstream tasks, such as speech recognition [8], machine translation [9] or medical abbreviation disambiguation [10].

A variety of word embedding models exist, among which the most popular ones focus on modeling words’ similarity based on their co-occurrences within local textual contexts. After the Word2Vec [11] and GloVe (Global Vectors for Word Representation) [12] models were introduced in 2013–2014, word embedding methods gained immense popularity. Two of the most popular models introduced afterwards are fastText [13] and BERT (Bidirectional Encoder Representations from Transformers) [14]. The **Word2Vec** [11] approach introduces two similar yet distinct models known as continuous bag-of-words (CBOW) and skip-gram (SG). It consists of training a shallow neural network to either predict a masked word based on its surrounding context window (CBOW) or predict probable neighboring words within the context window of a single input word (SG). In both cases, training samples are generated automatically from a large text corpus, and at the end, the weights of the model’s input layer are kept as the learned embedding matrix. **GloVe** [12] also applies a sliding context window to the training corpus but uses it to build a corpus-wide co-occurrence matrix, which is then factorized by solving a linear problem. The first of the smaller two matrices output by this factorization is kept as an embedding matrix. GloVe thus combines the central idea of Word2Vec, *i.e.* learning representations based on context, with a matrix factorization method that enables leveraging global co-occurrence statistics [15]. **FastText** [13] is a modification of Word2Vec that embeds subwords, *i.e.* substrings of words, in addition to entire words, therefore integrating subword information and internal structure of the words to improve the quality of their embeddings. **BERT** [14] is a more recently developed method that consists of training a deep neural network to predict randomly-masked words within input texts, and solve an optional text-coherence binary classification task. The encoder network follows the Transformer architecture, characterized by the use of self-attention mechanisms, so that output word encodings embed information from the entire input text. After pre-training, the entire encoder network is kept (only the pre-training-specific output layers are dropped), and can be fine-tuned on downstream tasks.

These models have been broadly applied in bioinformatics and healthcare. Many authors have applied them in a straightforward way, to learn embeddings of words or documents and/or perform downstream NLP tasks using clinical text data. A broad survey of such studies may notably be found in Kalyan & Sangeetha (2019) [15]. On the other hand, many methods have been developed and adopted to be used specifically on biomedical structured or unstructured data other than text. As a matter of fact, word embedding methods may also be applied to non-textual data that can be viewed as items belonging to a given vocabulary (acting as words) co-occurring within sequences (acting as sentences or documents). In bioinformatics, embedding methods have also been applied and adapted to embed –omics or molecular sequence data, which may be represented as strings containing subsequence patterns. For instance, frameworks such as **SPVec** [16], **Bio2vec** [17] and **IVS2Vec** [18] have been proposed to embed protein sequences data and use the resulting representations to predict drug-target interaction, and thus detect possible drug therapeutic targets or identify targets related to adverse drug relations.

On the same momentum, several methods have been adapted to healthcare data, of which we only name a few. **EHR2Vec**, for instance, is a method to process phenotypic information extracted from clinical notes that aims to identify relevant clinical concepts, taking into account time-sequence information from multiple visits [19]. **Med2Vec** is another method that aims to tackle the temporal nature of EHR data and to learn interpretable representations, which was initially applied to diagnostic, procedure and medication codes associated with patients’ visits [20]. **Snomed2Vec** used a clinical knowledge graph, SNOMED-CT, which constitutes an entirely distinct type of data structure, and learned Poincaré embeddings out of it, which the authors demonstrated to be efficient for predicting a patient’s condition [21]. Last, **Phe2Vec** is a disease-phenotyping framework that relies on an embedding learned over heterogeneous EHR data, including activity codes, vital signs, and selected terms parsed from clinical notes [22].

Notwithstanding the highly-variable specifics of the methods and applications, the common goal of learning embeddings from data in an unsupervised way is to produce representations that capture or reveal structural properties of the data as part of their geometry. Since embeddings project data in a low-dimensional numerical (most often, euclidean) space, they may be visualized in order to display those properties. Indeed, Zhang. Z (2019) highlighted how visualizing word embeddings helps specialized and non-specialized users of the models better discern the relations between the different features [23]. Further to this subject, Wang et al. (2018) reported that visualization might show different aspects of medical concepts captured by word embeddings trained from different corpora [24]. However, this approach appears to remain rather marginal; among the studies we cited up to this point, very few applied visualization tools on their embeddings [16, 22].

Currently, the most popular visualization methods and models are the following:

- t-SNE [25]: t-distributed Stochastic Neighbor Embedding is a probabilistic approach to the task of placing objects, described by high-dimensional vectors or by pairwise dissimilarities, in a low-dimensional space in a way that preserves neighbor identities. The t-SNE algorithm relies on constructing a probability distribution over pairs of points in a multi-dimensional space, designed to be proportional to the points' dissimilarity (*e.g.* the euclidean distance between them). First, this distribution is computed over the points in their original high-dimensional space, and each and every point is assigned random coordinates in the target low-dimensional map. Then, these coordinates are iteratively updated based on the objective to minimize the Kullback–Leibler divergence between the probability distributions computed in the high- and low-dimensional spaces.
- PCA [26]: Principal Components Analysis is a widely-used dimensionality reduction technique in data analysis that relies on finding orthogonal linear combinations of the initial feature dimensions that extract the most variance, and projecting the data points onto (a subset of) them. Its popularity notably comes from the fact that it is the optimal (with respect to mean squared error) linear scheme for compressing a set of high dimensional vectors into a set of lower dimensional vectors. Another valuable property is that the optimal solution is unique and can be reached exactly using spectral decomposition of the data’s variance–covariance matrix (although the cost for doing so may grow beyond reasonable for datasets with thousands of features).
- UMAP [27]: Uniform Manifold Approximation and Projection, is a novel manifold learning technique for dimension reduction. UMAP is constructed from a theoretical framework based on Riemannian geometry and algebraic topology. The result is a practical scalable algorithm that is applicable to real-world data. The UMAP algorithm is competitive with t-SNE for visualization quality, and arguably preserves more of the global structure with superior run time performance. Furthermore, UMAP has no computational restrictions on embedding dimension, making it viable as a general purpose dimension reduction technique for machine learning.
- K-Means [28]: although not a visualization technique per se, clustering (also known as unsupervised classification) methods may be leveraged to analyze embeddings and enhance their visualization. The K-Means clustering algorithm is straightforward to implement and can be applied to large and high dimensional data sets. It aims to partition n observations from a given data set (× 1,…, xn), where xi’s are d-dimensional vectors, into a predefined number of clusters k, denoted by (C1,…, Ck). This partition implies that each observation belongs to the cluster with the nearest center. K-Means optimization scheme consists of minimizing the sum of distances of each observation within clusters to the clusters’ centers. As a consequence, these clusters can be used to draw groups of similar elements based on their embedding representations, which can in turn be explored descriptively or displayed visually.

## Objective

This scoping review aims to describe the methods and strategies used to visualize medical concepts represented using word embedding methods. We aim to understand the objectives of the visualizations and their limits.

To the best of our knowledge, no review of the existing embedding visualization practices exists, nor are general guidelines available as to the way visualization methods may or should be used in conjunction to embeddings of clinical data.

Our paper is organized as follows: the search methodology, selection criteria, screening and analysis processes for the reviewed articles are presented in "Methodology" section. The included studies are analyzed in “Results” section and are discussed in “Discussion” section. Finally, the most relevant findings and the limitations of the study are summarized in “Conclusion” section.

## Methodology

### Overview

This scoping review summarizes different methods used to visualize word embeddings in healthcare. We conducted a scoping review using the methodology proposed by Arksey and O’Malley [29]. This scoping method is extensively used in scoping reviews related to healthcare. This framework suggests significant recommendations to compile findings and identify research gaps in the actual literature. We also took inspiration from the scoping methodology proposed by Levac et al. [30] for the data synthesis and the colleagues consultation sections.

### Search strategy

A literature review has been performed to determine relevant papers that used different visualization methods to represent their word embeddings. The search has been run from November 30th, 2020 until February 15th, 2021. The databases queried were PubMed, MedRxiv and arXiv. Due to the recent popularity of embeddings, we restricted our search to the papers which were published from January 2010 until January 2021. We focused on papers written in English only. The search string used in the different databases was modulated to get the highest number of relevant papers. For instance, the word “medical” is added to the search string of arXiv, a generalist database. Databases queries were as follows:

The search string for PubMed:

*visualization [All Fields] AND word [All Fields] AND embeddings [All Fields]*

The keywords used on MedRxiv:

*visualization word embeddings*

The keywords used on arXiv:

*visualization of medical word embeddings*

### Study selection criteria

In order to pick the relevant articles, we defined the following selection criteria: papers are written in the English language, papers are published from January 2010 up until January 2021, papers cover different types of studies: research papers, systematic reviews and case studies, papers are related to healthcare, papers include algorithms or models allowing to visualize the embeddings.

The articles were excluded if they were not written in the English language, did not include word embeddings nor visualize them and did not report information regarding technical aspects of the model/algorithm used to generate the embeddings and visualize them.

### Screening process

Following the search strategy, the selected papers’ screening process was performed independently by two authors (NO and SM). The process was divided into two phases. First, the authors reviewed the titles and abstracts resulting from the queries. Then, the authors analyzed all the manuscripts selected during the first step. The conflicts were resolved by common consensus.

### Data extraction

Data extraction from the included studies was performed by three authors (NO, SM, and AL). The extracted data was split into two groups, generic and technical information. Generic information involves the title, the abstract (objectives), the authors, the publication year, and the country of the first author.

The extracted technical information covered a variety of aspects. First, the main goal of the studies and the nature of the embedded data (words, codes…). Second, the embedding models used and their parameters, starting with dimensionality. Third, the evaluation methods and metrics used by the authors to assess the quality of their embedding models. Fourth, the origin, nature and sizes of the databases used to either learn or evaluate the embedding models. Finally, the visualization methods, their specifications, and their purpose as part of the studies.

### Data synthesis: collating, summarizing and reporting findings

This section has been proposed by Arksey and O’malley (2005) [29], and extensively detailed by Levac et al. (2010) [30]. They highlighted the importance of breaking the analysis phase into meaningful and systematic steps so that researchers can provide exhaustive scoping studies and report on findings in a rigorous manner. First, we performed a qualitative review and synthesis of the studies’ characteristics, with a focus on summarizing information on embedding models, data sources, data size, data types, and visualization methods. Second, we conducted a qualitative review and synthesis of the embedding method’s characteristics and of the different evaluation methods used. Third, we comprehensively synthesized all visualization efforts, focusing on their methodology and their aim. Finally, we discussed the pertinence of the studies’ findings and their implications for future research and practice.

### Presenting results to colleagues

Following the scoping methodology described by Levac et al. [30], we presented our results to colleagues interested in the use of word embeddings in healthcare, in order to benefit from their critical point of view and their expertise on this research topic.

## Results

**Fig. 1:**
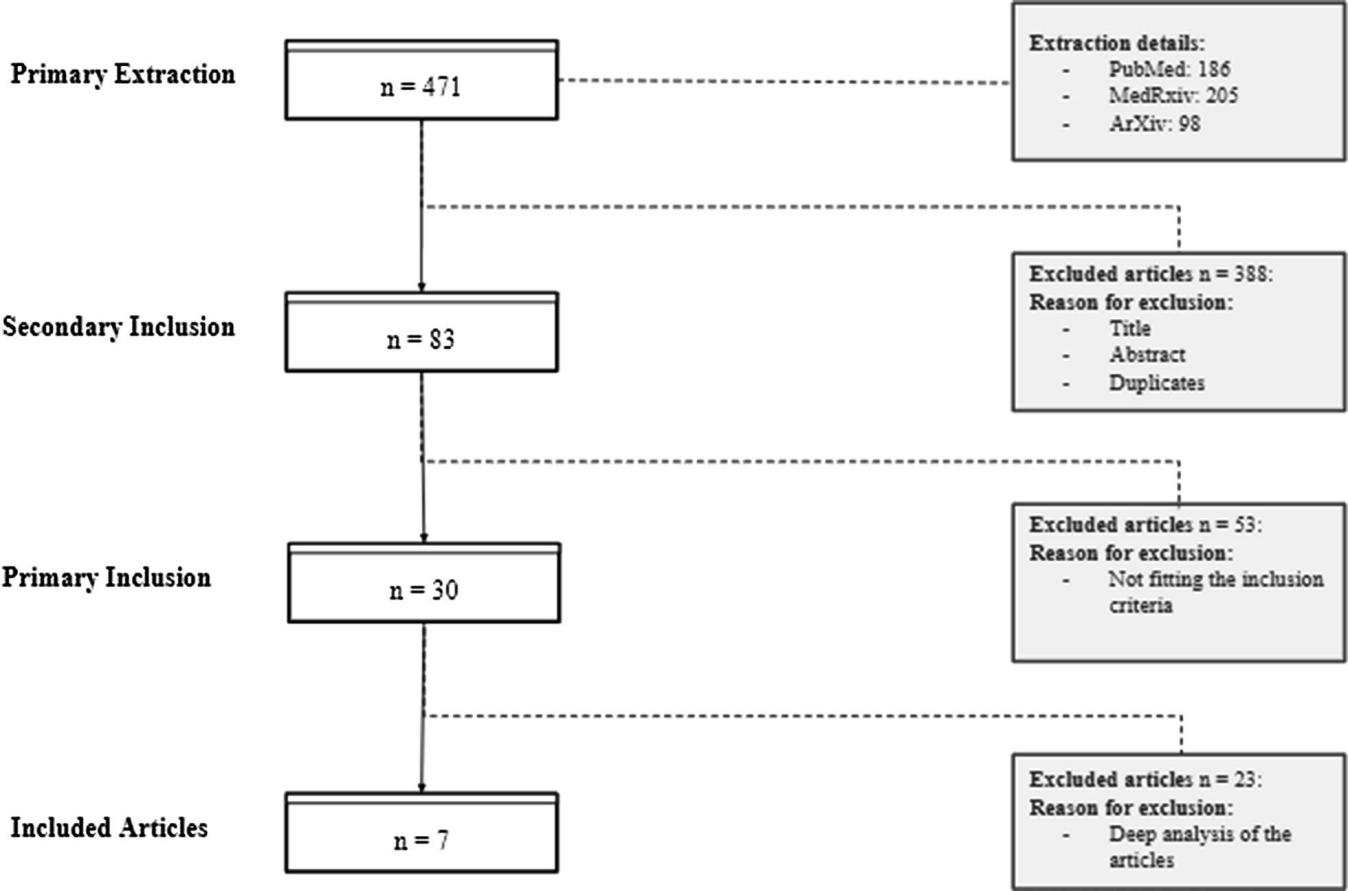
Articles selection process

We first retrieved 471 unique articles from the search conducted in research databases. 30 of these articles matched our primary inclusion and exclusion criteria. After their full review, 24 articles were excluded, due to their being either out of scope or of limited scientific interest. We notably ended up excluding nearly all articles retrieved solely from medrXiv or arXiv, with the exception of one [22], which has since been published in a PubMed-indexed peer-reviewed journal. This left us with 6 papers that fully satisfy to our inclusion criteria, plus one article which was added at a reviewer’s suggestion, leaving us with 7 articles to analyze. Details about the papers selection step are described below in Fig. 1.

### Description of the included studies

This section introduces a brief description of the articles included in this scoping review. All papers were published between 2018 and 2021.

Wang et al. [24] compared word embeddings learned from four different corpora: two generic ones (for which they used publicly-available pre-trained models), one made of biomedical publications, and one made of unstructured EHR data. They conducted a qualitative evaluation of the embeddings, as part of which they visualized their 60-, 100- or 300-dimensional embeddings using a 2-dimensional plot. They also conducted a two-fold quantitative evaluation, investigating both the intrinsic properties of the embeddings as to medical terms' similarity using external datasets, and the extrinsic benefit of using their embeddings as input representations to supervised machine learning models that perform some biomedical NLP tasks. They concluded that the word embeddings trained from EHR data are the most relevant in terms of in-domain intrinsic properties, but that they do not outperform embeddings learned from general data as input features to downstream tasks.

Shah et al. [31] proposed a concept association mining framework in order to model and analyze disease and symptom relationships in clinical notes. They learned word embeddings from clinical notes of the Indiana University Health, then focused on tokens that were categorized as diseases and symptoms by a third-party automated annotation tool. They computed the association matrix between those medical concepts using cosine distance, and performed a k-means clustering out of it so as to obtain 50 groups of symptoms and diseases. The authors then assessed the pertinence of the resulting clusters and terms associations through limited manual review. They also proposed a visualization method of patients’ diseases and related symptoms in chronological order, with time along the x-axis and grouping of items along the y-axis based on cluster assignment. The authors also reviewed some plots manually as part of their quality assessment of the embedding and clustering results.

Beaulieu-Jones et al. [32] applied Poincaré embeddings to represent ICD-9 diagnostic codes in a 2-dimensional hyperbolic space. They learned their embeddings from a large-scale insurance administrative claims database, and assessed the coherence between the obtained representations and the ICD-9 hierarchy using quantitative metrics. The same comparison was conducted with embeddings learned in euclidean spaces of varying dimensionality, and the authors concluded that their 2-dimensional Poincaré embeddings' coherence was on par with that of 100-dimensional euclidean ones. Finally, they provided visualizations to better illustrate the coherence with the ICD-9 hierarchy structure.

Dynomant et al. [33] learned a variety of word embedding models on a corpus of French-language text documents originating from the Rouen University Hospital, in order to compare embedding methods. They used five distinct evaluation tasks resolving around the intrinsic properties of the embeddings. Among these, they produced a 2-d visualization of each of the embeddings and manually reviewed so-called “visual clusters” emerging from them. Based on the other four tasks, which are more formal, they concluded that the embeddings produced by SkipGram Word2Vec had the best properties.

De Freitas et al. [22] introduced Phe2vec, an automated framework for disease phenotyping, which relies on learning a word-embedding model that encodes both selected terms extracted from clinical notes, vital signs, diagnoses, procedures, lab tests and medications codes in a single embedding space. To automatically define disease phenotypes, the authors extracted the ICD code associated with the disease and its closest embedding vectors. They produced a 2-d visualization of their embeddings, meant to illustrate the spatial distribution of those phenotypes. Then, they designed formulas to build synthetic representations of patients' EHRs and compute an association score between these and a given disease phenotype. To assess their framework’s effectiveness, the authors used Phenotype KnowledgeBase (PheKB), which is a collection of expert rule-based phenotyping algorithms. For each of ten selected diseases, they extracted the list of patients matching the PheKB criteria as gold standard, ranked all patients in their data using their approach and computed precision-at-recall to measure that ranking's pertinence. The authors concluded in a promising performance in automatic disease definition and patient cohort identification without the need for expert rules and definitions such as those from PheKB, which are effortful to produce.

Chen et al. [34] proposed an approach for evaluating the semantic relations in word embeddings using external knowledge bases: Wikipedia, WordNet [35] and Unified Medical Language System (UMLS) [36]. The authors aimed to elevate the transparency of word embeddings and the accountability of their applications. They evaluated the performance of their embeddings using analogy and semantic relation term retrieval tasks, and assessed the influence of the domains covered by the training textual corpora on the evaluation results.

El-Assady et al. [37] introduced a framework that allows users to incorporate domain knowledge to refine a topic model being learned from a corpus, the latent semantics of which rely on word embeddings as base representations. The authors proposed and demonstrated a system that makes use of an ad hoc interactive visualization tool to display word associations extracted as part of a topic model, and to gather user feedback refining the grouping of words into concepts. Those feedbacks and changes in concept definitions result in additional computations that alter the topic model, hence defining an interactive loop where users can visualize and trigger the evolution of the modeling. While this paper does not target clinical data directly, it implicitly addresses a number of generic and critical concerns regarding the way embeddings are evaluated and explored through visualization, and is illustrative of how they may be tackled with new methods. Therefore, we chose to include it as part of our review.

### Studies’ objectives

Among the included articles, we identified four key motives. Two studies [24, 34] aimed at comparing embeddings learned from distinct databases, so as to assess the impact of using in-domain data rather than generalist data. Two studies [22, 31] aimed to extract structured clinical knowledge from data using embeddings. Two articles [32, 33] primarily aimed at comparing distinct embedding methods, which was also a secondary concern in [22, 34]. Finally, one article [37] introduced a new methodology that enables incorporating user feedback to refine the semantics captured by a word-embedding-based topic model.

### Data collection

In total, 6 articles used healthcare data to train their models (6 out of 7) [22, 24, 31–34]. 4 of them worked on Electronic Health Records from hospital systems (4 out of 7) [22, 24, 31, 32], one used an administrative claims database (1 out of 7) [32], and one used healthcare articles from Wikipedia (1 out of 7) [34]. Clinical notes were used by 3 papers (3 out of 7) [22, 24, 31]. 2 articles used ICD-9 codes (2 out of 7) [22, 32], *i.e.* nomenclature-encoded information on diagnostics and medical procedures associated with individual hospital stays. Only one article [22] used both clinical notes and ICD-9 codes. 2 studies used scientific publications as training data (2 out of 7) [24, 33]. One of them used PubMed articles [24] and the other used French Abstracts from the Lissa Database [33].

Other data available on the internet, such as Wikipedia articles, were used by Wang et al. [24] and Chen et al. [34] (2 out of 7). All papers used the same data for training and testing except Wang et al. [24] who used data from Pedersen [38], Hliaoutakis [39], MayoSRS [40], and UMNSRS [41] to validate their results; and Chen et al. [34] who used WordNet [35] and UMLS [36] to evaluate the performance of their embeddings using term-retrieval tasks.

Most studies (4 out of 7) [22, 24, 31, 34] learned word embeddings from unstructured text data. Two studies [22, 32] applied word embedding methods to represent ICD-9 diagnostic codes, one of which jointly embedded other clinical activity and phenotyping codes as well as selected words from clinical notes.

The size of the training datasets ranged from 113 k patients to 63 M patients. Whereas the size of the test datasets ranged from 34 k words to 57 k clinical concepts from 4.5 M patients. The comparability of these numbers is limited, because the studies mobilize different types of data. All dataset characteristics are described in Table 1 (at the end of this article), along with the algorithms at stake and the corresponding data used for visualization.

**Table 1:** Detailed information on the data used in the studies: sources, sizes, types, and visualized features

Study	Algorithm/method	Training data (n = Corpus Size, d = Embedding Dimensionality)	Test data (size)	Visualized data
Wang et al. [24]	Word2Vec SG, Pretrained GloVe	Mayo Clinic clinical notes (n = 113 k patients: 103 k words, d = 100) MedLit Articles from PubMed Central [40] (n = 2 million words, d = 60) GloVe Wikipedia (n = 400 k embedded words, d = 100) Google News (n = 3 million embedded words, d = 300)	Pedersen [38] (NA) Hliaoutakis [39] (NA) MayoSRS [40] (NA) UMNSRS [41] (NA)	377 medical terms (symptoms and drugs) selected among all embedded ones
Shah et al. [31]	Word2Vec SG	The Indiana University Health’s EHR system (n = 500 patients: 154 738 clinical notes, d = 300)	The Indiana University Health’s EHR system (NA)	Patient-wise historical list of diseases and symptoms (assigned to 50 embedding-based clusters)
Beaulieu et al. [32]	Poincaré embeddings	Administrative claims database including diagnostic ICD-9 codes (n = 63 million patients, d = 200)	Administrative claims database including diagnostic billing ICD-9 codes (13.38 million patients)	All embedded ICD-9 diagnoses codes (223 million)
Dynomant et al. [33]	Word2Vec SG, Word2Vec CBOW, FastText SG, FastText CBOW, GloVe	Rouen University Hospital Data (641 279 documents) French medical paper abstracts from the LiSSa corpus (1.25 million of French abstracts)	Rouen University Hospital Clinical Data (607 135 health documents for Word2vec SG model)	All embedded words (50 066), with a focus on visually-extracted sub-regions
De Freitas et al. [22]	Word2Vec SG, FastText SG, GloVe	Medical concepts (diagnostics, procedures, lab tests and medication codes, plus selected words) extracted from structured EHRs and unstructured clinical notes from the Mount Sinai Health System (n = 2 208 741 patients: 49 234 medical concepts, d = 200)	De-identified EHRs from the MSHS data warehouse (validation: 4.5 million patients and 57 464 clinical concepts, testing: 1 608 741 patients)	All embedded medical concepts (57 464), with highlight of ten disease codes and their neighborhoods
Chen et al. [34]	Word2Vec, GloVe, Dependency-based word embeddings [42]	Wikipedia health-related articles (n = 322 339, d = 300)	Wikipedia general articles (NA), WordNet (9000 analogy questions), UMLS (33 000 analogy questions)	Relation terms of specific words and their top-10 nearest neighbors in the reduced 2-D space of the embedding space
El-Assady et al. [37]	LDA, pre-trained embeddings from ConceptNet [43]	Utterances from the 2nd Obama-Romney 2012 US presidential debate	NA	Words and their grouping into user- or model-defined concepts and topics

NA is used when the information is not available

The study performed by El-Assady et al. [37] used data from the 2^nd^ Obama-Romney US presidential debate in 2012 as an application case. Also, two studies did not provide information about the size of their testing databases [24, 31].

#### Algorithms

8 different embedding algorithms were used to implement the models proposed in the included studies. They include the two Word2Vec methods (CBOW / SG); the reciprocal two FastText ones; GloVe; Poincaré Embeddings; dependency-based embeddings [42] (which are derivative of Word2Vec SG); and ConceptNet embeddings [43] (pre-trained embeddings learned from a terms-relationship knowledge graph). The detail of their use in the studies is specified in Table 1.

### Evaluation methods and metrics

Although their objectives differ, all of the reviewed studies express the need to assess the quality of the learned embeddings, and/or of the knowledge derived from them. They present some commonalities, with 5 (out of 7) papers conducting at least one formal evaluation of the embeddings to control whether the geometrical relationships between embedded terms match with some external expert knowledge. However, the methods used to do so and the associated metrics are very diverse.

Three papers investigated the coherence of terms’ associations by focusing on specific, manually-rated examples:

- Wang et al. [24] and Dynomant et al. [33] used databases of manually-rated **term pairs**, and measured the **cosine similarity** between these pairs in the embedding space.
- Dynomant et al. [33] and Chen et al. [34] performed **analogy-based operations**, *i.e.* they used manually-rated duplets of term pairs and measured to what extent the vector connecting the first two terms matched that connecting the other two.
- Dynomant et al. [33] computed the **odd one out similarity**, which measures the discriminability of an unrelated term from a pair of related ones based on their embedding vectors.
- Chen et al. [34] used databases that list words related to a word of reference, and computed the **retrieved-words ratio** in the neighborhood of the latter in the embedding space.
- Dynomant et al. [33] went the other way around, having human evaluators review lists of nearest-neighbors of terms of interest in the embedding space, and computing **formal pertinence metrics** as to those associations.

Two papers compared their embeddings to a current expert knowledge base in a more systematic manner, with the aim of extending or replacing it:

- Beaulieu et al. [32] assessed whether their ICD-9 embeddings **matched the ICD-9 hierarchy**, using a **distance-ratio** metric (within-group / out-of-group).
- De Freitas et al. [22] used their framework to target patients with a given disease phenotype and measured **precision-at-recall of patient retrieval** as compared with expert algorithms.

Three papers mention having human experts perform some reviewing, but do not provide any insight on a precise methodology or metric, which lets us believe that this **partial review** constitutes merely a **soft validation**:

- Shah et al. [31] mentioned reviewing the closest neighbors to some reference terms, as well as the closest-to-center terms of the clusters they built from their embeddings, but acknowledged how partial such a review is – and how tedious expanding it would be.
- El-Assady et al. [37] presented an application case for their algorithm, but only stated that the feedback loop they implemented resulted in improvements of their topic models’ pertinence, without any further details.
- Dynomant et al. [33], which otherwise conducted four formalized evaluations, listed as a fifth evaluation the manual investigation of “visual clusters” among the 2-d t-SNE plot of their embeddings. If the authors provide some insight on how results differ from model to model, they however do not elaborate on any solid formalized conclusion. We note that Wang et al. [24] adopted a similar data exploration approach through visualization, but did not describe it as an evaluation method.

Finally, one paper had a utilitarian approach to evaluate their embeddings, based on their leveraging so as to perform a downstream task:

- Wang et al. [24] investigated how substituting them as **input features to supervised machine learning** models performing a variety of information retrieval tasks **altered the models’ performance**, as measured using precision, recall and f1-score.

### Visualization

In this section we synthesize our findings on the visualization methods used in the reviewed articles and on their purposes as featured by the authors. Table 2 (at the end of the paper) also provides a summary of this information for each article.

**Table 2:** The different visualization methods and the visualization objectives

Study	Visualization objective	Visualization method
Wang et al. [24]	Exploration of «visual» clusters	t-SNE with focus on manually-selected areas
Shah et al. [31]	Synthesis of patients' medical history + soft validation of clusters, hence embeddings	ad hoc chronology of symptoms and diseases, organized visually based on embeddings’ cluster assignments
Beaulieu et al. [32]	Illustration of the embeddings’ consistency with the ICD-9 hierarchy	Plot of 2-d embeddings and Hierarchy Tree [44]
Dynomant et al. [33]	Exploration of « visual» clusters presented as an evaluation of the embeddings	t-SNE with focus on manually-selected areas
De Freitas et al. [22]	Illustration of the embeddings and of some identified disease phenotypes	UMAP with highlight of neighborhoods around selected diagnostic codes
Chen et al. [34]	Illustration of the embeddings and some relations between terms used as part of the evaluation tasks	PCA with highlight of selected words, some with their neighborhoods (used for word-retrieval evaluation)
El-Assady et al. [37]	Topic models’ exploration & user feedback gathering (part of the modeling process)	ad hoc t-SNE-based visualization of topics and related words grouped into concepts + interactive system to gather feedback and trigger model evolutions

#### Objective of the visualizations

We find that in all studies, visualization is leveraged to assess the data structure properties reflected in the geometrical properties of the learned embeddings. We note however that depending on the articles, authors either aim to assess the consistency of those properties with expected ones, or to illustrate relevant newly-found relationships identified thanks to the embeddings. Accordingly, we identified the following three archetypal groups, each describing a specific application of visualization as part of their method’s evaluation process:

##### Evaluation of the embeddings

Wang et al. [24] and Dynomant et al. [33] both visualized their embeddings in order to manually identify and explore “visual clusters”. As noted before, this rather-informal approach to explore and soft-validate the embeddings is only deemed to constitute an evaluation in the second of those articles.

Shah et al. [31] developed a patient-history visualization tool that organizes the displayed information based on a K-Means clustering derived from the embeddings, thus making the latter a mere instrument to the visualization purpose. However, the authors present this visualization effort as a component in their (soft) evaluation of the pertinence of the diseases-and-symptoms clusters derived from their embeddings.

##### Illustration of embeddings’ properties

Beaulieu et al. [32], De Freitas et al. [22] and Chen et al. [34] used the visualizations of their embeddings to illustrate information-grouping and information-retrieval properties they otherwise investigated formally using metrics computed from structured manually-rated knowledge databases. As such, we can consider their visualizations as a mere illustration of otherwise formally-assessed properties of their embeddings, focused on a limited number of examples and/or on the overall impression one can take out from plots that comprise thousands of points.

##### Interactive feedback system

El-Assady et al. [37] developed a two-fold interactive visualization system, which forms the core of their paper and bridges both illustration and evaluation objectives. Indeed, this system, together with an adaptation of topic models relying on word embeddings, aims to both display topic-modeling results and gather user feedback in order to constrain and improve the semantic properties of the learned model.

#### Visualization methods

All studies produced visualizations in the form of 2-dimensional plots. To do so, most studies (4 out of 7) used a dimensionality reduction method to project their multi-dimensional embeddings on a 2-dimensional space: Wang et al. [24] and Dynomant et al. [33] used t-SNE (which is also instrumental in the method developed by El-Assady et al. [37]), De Freitas et al. [22] used UMAP, and Chen et al. [34] used PCA. On the other hand, Beaulieu et al. [32], having produced 2-dimensional Poincaré embeddings, displayed them on their native, hyperbolic plane. Finally, Shah et al. [31] and El-Assady et al. [37] developed ad hoc visualization tools that organize the relevant information retrieved or derived from embeddings, rather than to display the embeddings themselves.

We also note that all direct visualizations of embeddings reported as part of the articles make use of some data selection or highlighting process. Wang et al. [24] and Dynomant et al. [33] provided a few manually-selected regions rather than the entire t-SNE plot. De Freitas et al. [22] and Chen et al. [34] highlighted some specific points among their whole visualized set, notably the closest neighbors of a few illustrative targeted elements. Beaulieu et al. [31] colored the embedded ICD diagnostics based on the ICD-9 hierarchy, and displayed hierarchy trees [44] on subsets of codes, illustrating structural properties otherwise quantified in the article.

## Discussion

### Synthesis of work

This scoping review intended to provide an overview of the uses of visualization methods applied to embeddings learned from healthcare data. We subsequently included 7 papers that reported word embedding graphical representation, with various methods and purposes. We reported the overall objectives of those studies, their methodology to produce and/or use embeddings, the way they evaluated the latter, the methods used to visualize the embedded data and the role of visualization in the study.

We initially retrieved a large number of articles from our search queries (471), but only a fraction passed our inclusion criteria (30), out of which only 7 were included for review after in-depth screening. This number is mostly due to the relative marginality of visualization in scientific publications about embeddings of clinical data. It is also not atypical for a scoping review, which consists of an in-depth analysis of selected papers with the objective to offer a qualitative analysis of practices rather than offer an exhaustive report as in a systematic review.

We note that in spite of our search targeting publications from 2010 and onwards, the included papers were in fact published between 2018 and 2021. Similarly, while our primary inclusion criteria made room for non-peer-reviewed articles retrieved from arXiv and medRxiv, we ended up discarding most such papers based on relevance and quality issues. Anecdotally, the only non-peer-reviewed article we included ended being published in a peer-reviewed, Pubmed-indexed journal during the revision phase of our work.

### Synthesis of findings

#### Embedding and evaluation practices

As largely detailed in the results section, and as denoted by the synthetic information presented in Tables 1 and 2, the included studies are heterogeneous in a number of ways. Embeddings were learned using 8 distinct methods, on different types of data (raw texts; selected terms extracted from texts; structured EHR data such as diagnostic or activity codes) from a wide range of in-domain and general sources, notwithstanding the use of pre-trained embeddings, notably for comparison purposes. The purposes of the studies were also diverse. Some authors conducted a methodological review of embedding methods. Others focused on various targeted uses of learned embeddings to extract structured knowledge from the data and/or perform downstream tasks, most of which revolved around information retrieval.

Evaluation of the learned embeddings or of the derived knowledge was an important topic in all of the reviewed papers, again with heterogeneous practices. A variety of metrics and reference information were considered by the studies’ authors. However, we found that they mostly investigated the concept associations drawn by the geometrical properties of the embeddings, with varying degrees of scope width. Some conducted systematic comparisons to expert in-domain knowledge bases; some considered subsets of specific, manually-rated examples with sizes ranging for a handful of cases to hundreds or thousands of concepts; and some only reported overall quality assessments based on non-systematic, non-quantified manual reviewing of a few examples. We note that only one paper mediated the evaluation of the embedding matrix by observing how leveraging it to reformat input features of a machine learning model may improve the latter’s achieved performance. We believe that the under-representation of this otherwise common use of embeddings in the papers we reviewed is coherent with our focus on visualization, as researchers interested in leveraging embeddings may be less eager to investigate their intrinsic properties than to conclude as to their utility with respect of their target downstream task.

#### Visualization methods and purposes

We remarked that visualization methods were highly heterogeneous between papers, but always resulted in 2-d plots. The latter were achieved either by using a further dimensionality reduction technique, having natively produced 2-d embeddings, or proposing an ad hoc visualization system organizing information based on computed geometrical properties.

We analyzed that in the reviewed papers visualization was always related to the evaluation of geometrical properties of the embeddings, or of the derived concept associations. We concluded however that the relationship between visualization and evaluation implied by the authors could go in opposite directions. Therefore, we proposed to regroup the purposes of visualization in the reviewed papers into three archetypal categories: evaluating the embeddings’ properties’ pertinence (often with limited insight on the exact protocol and metrics); illustrating properties after assessing them through computations (hence representing known information); or proposing an interactive feedback system that binds both previous purposes so as to allow users to review the visualized properties and to automatically derive formal constraints out of it.

## Analytical discussion

### Interest in visualizing embeddings

Even though embeddings are widely used in NLP tasks, they remain ambiguous. Embedding methods aim at capturing syntactic and semantic information, are well-grounded mathematically and have been shown empirically to yield results that are useful for both data exploration and transfer into downstream prediction tasks. However, assessing the quality and pertinence of a given learned embedding is rarely straightforward. As a matter of fact, it is not clear what exact properties are encoded in a given learned embedding matrix, which is why similarity-ranking and visualization of the embedded elements are often leveraged to explore it. This is a crucial stake, since with poor interpretability, embeddings cannot be used in applications where reasoning for decisions is required.

High dimensionality is one of the factors that can limit human apprehension of an embedding. Thus, using dimensionality reduction methods such as t-SNE and PCA to visualize embeddings makes it easier to apprehend the way the embedded elements are distributed and positioned relatively to each other within the embedding euclidean space. This can therefore help understanding and assessing the captured latent semantic properties of those elements. These visualization methods and others can be very useful when applied on medical/clinical embeddings as they can provide a synthetic view on the embedded healthcare data, possibly making it easier to understand to the specialized and non-specialized public.

### Limits of visualization

Based on the papers we reviewed, we remark that visualizing embeddings is alternatively presented as a way to validate the embedding’s consistency with pre-existing domain knowledge, or as a way to extract domain knowledge based on an embedding that is therefore implicitly trusted to be meaningful. This two-fold nature of data exploration is not exclusive to embedding techniques, but in our opinion, it conveys a risk of insufficiently evaluating an embedding, which can result in prematurely reifying it into knowledge. We believe that visualization, while very useful in exploring a learned embedding, does not constitute by itself a sufficient approach to assess the properties and pertinence of said embedding, at least not when limited to a single 2-d projection of a high-dimensional embedding.

As a matter of fact, while visualization may highlight some geometric similarity properties of the data, it is highly doubtable that anyone will review exhaustively the relative positions of the projected data points, so that while overall tendencies and/or specific examples may be efficiently investigated thanks to it, a visual review will most probably never be as systematic as similarity-ranking or matrix spectral analysis may be.

This is all the more true as dimensionality reduction techniques, which are required to visualize high-dimensional embeddings on a 2-d or 3-d plot, imply some information loss, the level of which is highly dependent on the properties of the initial matrix—something that none of the papers we reviewed ever mentions nor quantifies, in spite of it being very easy to do. For PCA, the percentage of entropy summarized by the axes used for visualization should be reported [26], while other metrics could be used for all the other methods, such as the Kullback–Leibler divergence or the euclidean norm of the difference between matrices of pairwise distances in the embedding and visualization space [45].

### Overall problem of evaluation

We are however convinced that a multi-dimensional exploratory analysis of a learned embedding, possibly helped by multiple, complementary visual representations (*e.g.* projections on pairs of PCA axis that altogether capture the greater share of the initial embedding matrix’s variance), can be a pertinent way to make sense of said embedding and of its properties. We believe that this can be all the more useful in cases when there is little or no prior knowledge to be leveraged into evaluating elements’ similarity, and therefore an actual sense in trying to unveil knowledge thanks to the embedding representations.

Our review highlights that there often is a lack of clear reference material to evaluate the learned embeddings. While all papers report computing and ranking elements’ similarity (using either euclidean or cosine distance), the way an evaluation is conducted based on those metrics differs greatly from paper to paper, with a minority of them making use of a formal standard to which results may be compared, and a majority involving some sort of qualitative assessment, with various levels of formalization and exhaustiveness. This issue of not having a clear and formal reference to compare to is unlikely to be easily solved, and is somehow the motivation for learning an embedding using unsupervised of self-supervised methods. Indeed, had we a knowledge base defining the latent properties we wanted to embed, we would instead make use of a metric learning method to learn an embedding constrained to capture them [45].

This is not necessarily a pitfall as embeddings are often used in an instrumental fashion, as a way to transfer knowledge for supervised tasks; in which case they can be evaluated by measuring the performance gains they yield in comparison with a baseline model, as was done in two of the papers we reviewed. Simply, one may perhaps gain in clarity defining whether the aim of the embedding is instrumental or exploratory; whether it should model knowledge to be leveraged by an algorithm that has the ability to make use of its high dimensionality, or to be explored and explained by a human who wants to extract target domain knowledge.

### Study limitations

While carrying out this review, we initially encountered a massive number of articles on different databases treating of word embeddings in general. We then decided to exclude BioArxiv and Google Scholar as data sources. During the data extraction phase, we were also confronted to a majority of retrieved papers that did not actually fall under our topic on visualization, which underlined the difficulty in defining a very stringent search query. Another study limitation is the restrained access to the raw data and graphical representations used in studies. We essentially based our analyses on what was disclosed in the papers by the authors, which may not fully reflect their actual data exploration efforts.

### Implications for research and practice

Our review reasserts the fact that embedding methods are a pertinent and popular way to learn structured representations of complex unstructured and/or high-dimensional raw data such as clinical texts or activity codes pertaining to very wide nomenclatures. Our specific focus on visualization of such embeddings highlights that visual representations have a potential to help in apprehending and exploring learned embeddings. Our work also underlines the benefit that may come from future research focused on providing shared guidelines for embeddings visualization, as well as new methods that bind exploratory and evaluative purposes together.

## Conclusion

The review aimed to widen our understanding about the usefulness of word embedding visualization. The review described 7 studies reporting the use of visualization for the evaluation of embedding models, for the exploration of large and multimodal datasets, and information retrieval. Visualization helps exploring embedding results (further dimensionality reduction, synthetic representation). However, it does not exhaust the information conveyed by the embeddings nor constitute a self-sustaining evaluation method of the latter’s pertinence.

## Data Availability

Not applicable.

## Abbreviations

BERT: Bidirectional encoder representations from transformers
CBOW: Continuous bag of words
EHR: Electronic health records
GloVe: Global vectors for word representation
GMM Clustering: Gaussian mixture models clustering
HAN: Hierarchical attention network
ICD-9: International Classification of Diseases, 9th Revision
LDA: Latent Dirichlet allocation
LSTM: Long short-term memory
MSHS: Mount Sinai health system
NLP: Natural language processing
PCA: Principal component analysis
SG: Skip-gram
SMILES: Simplified molecular-input line-entry system
t-SNE: T-distributed Stochastic neighbor embedding
UMAP: Uniform manifold approximation and projection
